# Antiproliferative Effects of *Cynara cardunculus* L. var. *altilis* (DC) Lipophilic Extracts

**DOI:** 10.3390/ijms18010063

**Published:** 2016-12-29

**Authors:** Patrícia A. B. Ramos, Ângela R. Guerra, Olinda Guerreiro, Sónia A. O. Santos, Helena Oliveira, Carmen S. R. Freire, Armando J. D. Silvestre, Maria F. Duarte

**Affiliations:** 1 Centro de Biotecnologia Agrícola e Agro-Alimentar do Alentejo (CEBAL)/Instituto Politécnico de Beja (IPBeja), 7801-908 Beja, Portugal; patricia.ramos@cebal.pt (P.A.B.R.); angela.guerra@cebal.pt (A.R.G.); olinda.guerreiro@cebal.pt (O.G.); 2 CICECO and Department of Chemistry, University of Aveiro, 3810-193 Aveiro, Portugal; santos.sonia@ua.pt (S.A.O.S.); cfreire@ua.pt (C.S.R.F.); armsil@ua.pt (A.J.D.S.); 3 CIISA, Centro de Investigação Interdisciplinar em Sanidade Animal, Faculdade de Medicina Veterinária, ULisboa, Avenida da Universidade Técnica, 1300-477 Lisboa, Portugal; 4 CESAM and Department of Biology, University of Aveiro, 3810-193 Aveiro, Portugal; holiveira@ua.pt

**Keywords:** *Cynara cardunculus* L. var. *altilis* (DC), lipophilic extracts, cynaropicrin, taraxasteryl acetate, triple-negative breast cancer MDA-MB-231 cell line, p21^Waf1/Cip1^ upregulation, phospho(Tyr15)-CDK1 protein accumulation, phospho(Ser473)-Akt downregulation

## Abstract

Besides being traditionally used to relieve hepatobiliary disorders, *Cynara cardunculus* L. has evidenced anticancer potential on triple-negative breast cancer (TNBC). This study highlights the antiproliferative effects of lipophilic extracts from *C. cardunculus* L. var. *altilis* (DC) leaves and florets, and of their major compounds, namely cynaropicrin and taraxasteryl acetate, against MDA-MB-231 cells. Our results demonstrated that MDA-MB-231 cells were much less resistant to leaves extract (IC_50_ 10.39 µg/mL) than to florets extract (IC_50_ 315.22 µg/mL), during 48 h. Moreover, leaves extract and cynaropicrin (IC_50_ 6.19 µg/mL) suppressed MDA-MB-231 cells colonies formation, via an anchorage-independent growth assay. Leaves extract and cynaropicrin were also assessed regarding their regulation on caspase-3 activity, by using a spectrophotometric assay, and expression levels of G2/mitosis checkpoint and Akt signaling pathway proteins, by Western blotting. Leaves extract increased caspase-3 activity, while cynaropicrin did not affect it. Additionally, they caused p21^Waf1/Cip1^ upregulation, as well as cyclin B1 and phospho(Tyr15)-CDK1 accumulation, which may be related to G2 cell cycle arrest. They also downregulated phospho(Ser473)-Akt, without changing total Akt1 level. Cynaropicrin probably contributed to leaves extract antiproliferative action. These promising insights suggest that cultivated cardoon leaves lipophilic extract and cynaropicrin may be considered toward a natural-based therapeutic approach on TNBC.

## 1. Introduction

Breast cancer represents the most prevalent cancer among women, and one of the most frequent causes of female cancer death [1]. Triple-negative breast cancer (TNBC) is characterized by estrogen receptor (ER) and progesterone receptor (PR) negative, and lack of human epidermal growth factor receptor type 2 overexpression, accounting for 10%–20% of breast cancer cases. So far, targeted therapy was not found for TNBC [2]. The most common chemotherapy lies on primary anthracycline and anthracycline/taxane derivatives, which generally is not efficient since it carries high relapse risk during the first three years after treatment, and high incidence of metastases in the liver, central nervous system, and lungs [3]. The development of new TNBC therapeutic strategies, as preventive or complementary approaches, seems to be of high importance, especially in a scenario of limited therapeutic options, with several side effects and short survival rates. A decreased breast cancer risk has been associated with a diet rich in vegetables and fruits, in part due to the benefits of ω-3 fatty acids, carotenoids, phytosterols, and phenolic compounds [4,5,6,7]. Phytochemicals can indeed be considered as key agents for chemoprevention, as well as for new or complementary approaches to chemotherapy, based on their reduced toxicity, desired efficacy, and pleiotropic mechanisms of action [8].

The Mediterranean species *Cynara cardunculus* L. (Asteraceae) comprises three varieties, namely *sylvestris* (Lamk) Fiori (wild cardoon), *scolymus* (L.) Fiori (artichoke), and *altilis* (DC) (cultivated cardoon). Cardoon capitula are used in the making of Iberian traditional cheeses [9], whereas blanched cultivated cardoon leaf petioles are quite appreciated in Spanish, Italian, and French gastronomy [10]. Moreover, *C. cardunculus* L. leaf infusions are well-known in the folk medicine, owing to their hepatoprotective [11], choleretic, and anticholestatic actions [12,13]. These health-promoting effects have been associated with the presence of phenolic compounds, such as hydroxycinnamic acids (e.g., 1,3-di-*O*-caffeoylquinic acid) [11] and flavonoids (e.g., luteolin and luteolin 7-*O*-glucoside) [13]. Antioxidant [14,15], anti-inflammatory [16] and antimicrobial activities [17,18] of *C. cardunculus* L. extracts have also been noticed.

*C. cardunculus* L. extracts have demonstrated in vitro antiproliferative potential against breast cancer [15,19]. An artichoke capitula methanol extract prevented the 24 h cell growth of several breast cancer cell lines, namely MDA-MB-231 (TNBC), T47D (ER positive) and BT549 (ER negative) cells, in a dose-dependent manner. Additionally, this extract evidenced apoptotic effect in MDA-MB-231 cells, which may be mainly ascribed to phenolic compounds as, for instance, 5-*O*-caffeoylquinic acid [19]. Our research group also reported that the 48 h exposure of MDA-MB-231 cells to increasing concentrations of wild cardoon and cultivated cardoon hydromethanol extracts decreased their cellular viability [15].

**Figure 1:**
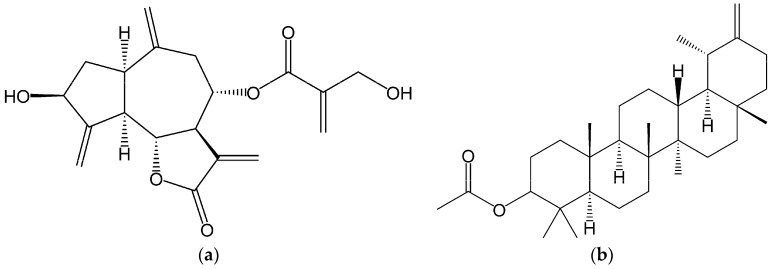
Structures of major lipophilic compounds identified in *C. cardunculus* L. var. *altilis* (DC) leaves and florets: (**a**) Cynaropicrin; (**b**) Taraxasteryl acetate.

In addition to phenolic compounds [20], cultivated cardoon leaves and florets are a source of sesquiterpene lactones (≈94.5 g/kg dry weight (dw)) and pentacyclic triterpenes (≈27.5 g/kg·dw), mainly represented by cynaropicrin (≈87.4 g/kg·dw of leaves) and taraxasteryl acetate (≈8.9 g/kg·dw of florets), respectively [21] (Figure 1). Cynaropicrin and taraxasteryl acetate have displayed anticancer potential against breast cancer, via in vitro [22] and in vivo [23] models. In fact, our research group previously reported that cultivated cardoon leaves dichloromethane extract and cynaropicrin inhibited MDA-MB-231 cell growth, by affecting cell cycle and Akt signaling pathway [22]. Additionally, an increased survival ratio of C3H/OuJ mammary tumor bearing mice was found after ingestion of taraxasteryl acetate [23].

In the scope of our searching for phytochemical-based targeted TNBC therapeutic [15,22,24], the present work aims to understand the inhibitory actions of cultivated cardoon leaves and florets lipophilic extracts, on MDA-MB-231 cellular proliferation. Pure cynaropicrin and taraxasteryl acetate, representing the most abundant compounds of extracts, were also studied. Based on the IC_50_ values, we will choose one extract and its major compound to assess their effects on: (i) tumor cell colonies formation; (ii) caspase-3 activity; (iii) cells distribution in G0/G1, S and G2 cell cycle phases; (iv) protein expressions of G2/mitosis checkpoint markers; and (v) protein expressions of Akt pathway markers.

## 2. Results

### 2.1. Inhibitory Effects of Cultivated Cardoon Lipophilic Extracts and Its Major Compounds on MDA-MB-231 Cellular Viability

Considering the preliminary insights of TNBC antiproliferative potential exhibited by cultivated cardoon extracts [15,22], we determined the IC_50_ values of cultivated cardoon leaves and florets lipophilic extracts, upon MDA-MB-231 cellular viability after 48 h, via MTT assay (Table 1). The inhibitory effects of the major extracts compounds, namely cynaropicrin and taraxasteryl acetate, were also determined.

The IC_50_ value of leaves lipophilic extract was 30-fold lower than of florets lipophilic extract (10.39 and 315.22 μg/mL, respectively) (*p* < 0.0001). Pure cynaropicrin, representing the major component of leaves lipophilic extract, also suppressed the MDA-MB-231 cell viability, with an IC_50_ of 6.19 µg/mL (17.86 ± 1.65 µM). Pure taraxasteryl acetate, representing the most abundant compound of florets lipophilic extract, was not tested at the same concentration range of cynaropicrin, due to solubility issues. At the range 0.05–2.34 μg/mL (or 0.1–5.0 µM), this pentacyclic triterpene did not decrease the MDA-MB-231 cellular viability during 48 h.

We also observed that human breast epithelial MCF-10A cells were sensitive to cultivated cardoon leaves lipophilic extract for 48 h, showing an IC_50_ of 11.29 ± 2.48 µg/mL which was not significantly different from the IC_50_ for MDA-MB-231 cells (*p* > 0.05).

### 2.2. Effects of Cultivated Cardoon Leaves Lipophilic Extract and Cynaropicrin on MDA-MB-231 Cells Colonies Formation Using Anchorage-Independent Growth Assay

**Figure 2:**
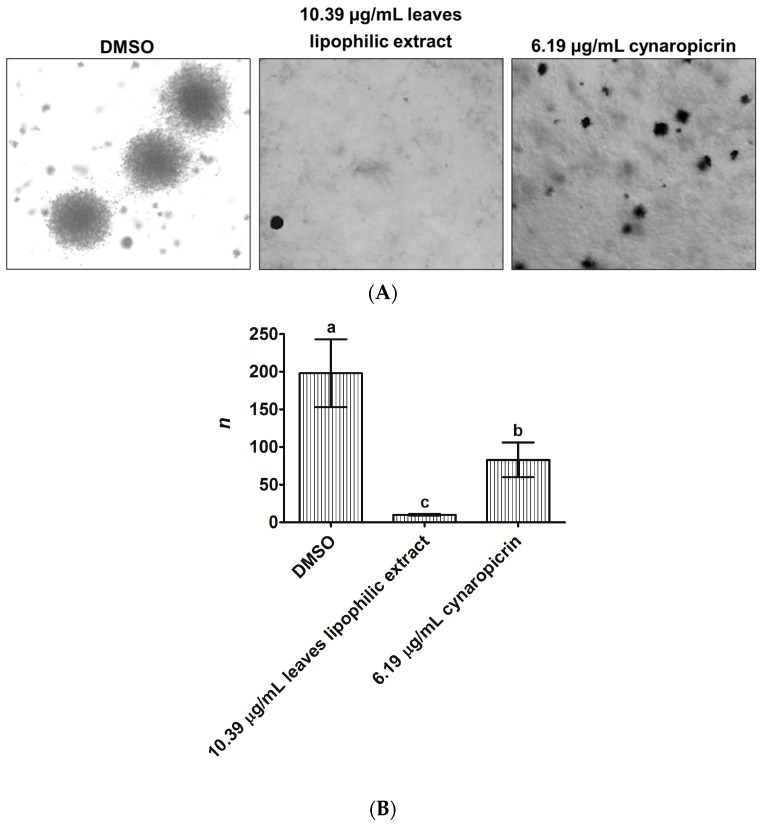
Effect of 10.39 µg/mL cultivated cardoon leaves lipophilic extract and 6.19 µg/mL cynaropicrin, against MDA-MB-231 cells colonies formation, via an anchorage-independent growth assay, after 14 days. DMSO (0.09% (*v*/*v*)) was used as solvent control. (**A**) Representative photographs of colonies (40× magnification); (**B**) Number of colonies (*n*). Each column and bar represents the mean and the standard deviation, respectively. Triplicates were carried out. Columns with different letters are statistically different (*p* < 0.05).

We sought to know whether cultivated cardoon leaves lipophilic extract and cynaropicrin could prevent the formation of MDA-MB-231 cells colonies for 14 days, in a three-dimensional environment, by using the anchorage-independent growth assay (Figure 2A,B).

Cultivated cardoon leaves lipophilic extract (10.39 µg/mL) and cynaropicrin (6.19 µg/mL) reduced the number and size of MDA-MB-231 cells colonies, relatively to dimethyl sulfoxide (DMSO)-control (Figure 2A). MDA-MB-231 cells thus lost their capacity to generate colonies (Figure 2B), especially when exposed to leaves lipophilic extract (19.8-fold vs. DMSO (*p* < 0.0002)) which was more effective than cynaropicrin (8.3-fold (*p* < 0.0215)).

### 2.3. Analysis of Caspase-3 Activity in MDA-MB-231 Cells after Exposure to Cultivated Cardoon Leaves Lipophilic Extract and Cynaropicrin

**Figure 3:**
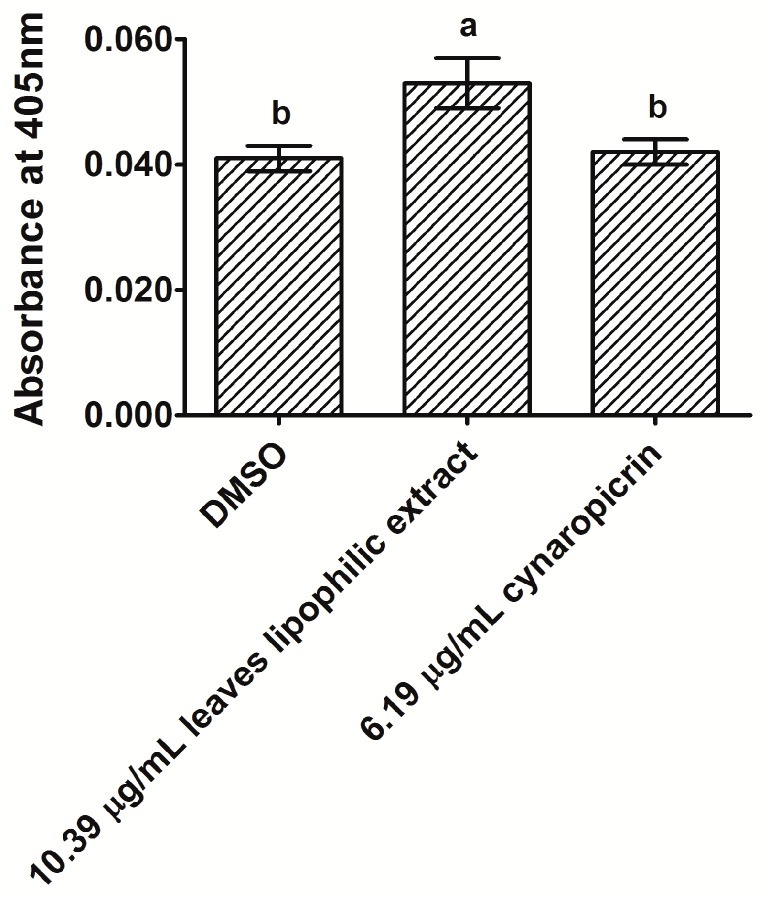
Caspase-3 activity assessment in MDA-MB-231 cells, after 48 h treatments with 10.39 µg/mL cultivated cardoon leaves lipophilic extract and 6.19 µg/mL cynaropicrin. DMSO (0.09% (*v*/*v*)) was used as solvent control. Each column and bar represents the mean and the standard deviation, respectively. Three independent experiments were carried out. Columns with different letters are statistically different (*p* < 0.05).

We researched whether cultivated cardoon leaves lipophilic extract and cynaropicrin could cause apoptosis in MDA-MB-231 cells, by examining caspase-3 activity via a commercial colorimetric kit (Figure 3).

After 48 h of incubation, leaves lipophilic extract (10.39 µg/mL) increased caspase-3 activity in MDA-MB-231 cells (1.3-fold vs. DMSO control (*p* < 0.0013)). On the contrary, the activity of this enzyme was not significantly affected in MDA-MB-231 cells treated with 6.19 µg/mL cynaropicrin, relatively to DMSO control cells (*p* > 0.05). More investigation is required to clarify whether cultivated cardoon leaves lipophilic extract and cynaropicrin might induce apoptosis in MDA-MB-231 cells.

### 2.4. Effects of Cultivated Cardoon Leaves Lipophilic Extract and Cynaropicrin in MDA-MB-231 Cells Distribution through Cell Cycle Phases

We previously observed that cultivated cardoon leaves dichloromethane extract and cynaropicrin could stop the MDA-MB-231 cell cycle progression at G2 phase [22]. Here, we analyzed the MDA-MB-231 cells distribution through cell cycle phases (G0/G1, S and G2), via flow cytometry, after 48 h of incubation with cultivated cardoon leaves lipophilic extract and cynaropicrin (Table 2).

Cultivated cardoon leaves lipophilic extract (10.39 μg/mL) led to a significant accumulation of MDA-MB-231 cells at G2 phase (Table 2), representing a 4.7-fold increment of cell percentage compared with DMSO control (*p* < 0.0001). There was a corresponding decrease of cells percentage at G0/G1 and S phases (2.7- and 1.8-fold vs. DMSO, respectively). Pure cynaropicrin (6.19 µg/mL) also caused MDA-MB-231 cell cycle arrest at G2 phase (Table 2), but in a less extension (3.0-fold increase of cell percentage relatively to DMSO control (*p* < 0.0001)). Moreover, cynaropicrin reduced the S phase cell percentage up to 2.1-fold (*p* < 0.0001), but it did not significantly alter the cell percentage at the G0/G1 phase, regarding to DMSO control (*p* > 0.05).

### 2.5. Effects of Cultivated Cardoon Leaves Lipophilic Extract and Cynaropicrin on p21^Waf1/Cip1^, Phospho(Tyr15)-CDK1 and Cyclin B1 Protein Expressions in MDA-MB-231 Cells

**Figure 4:**
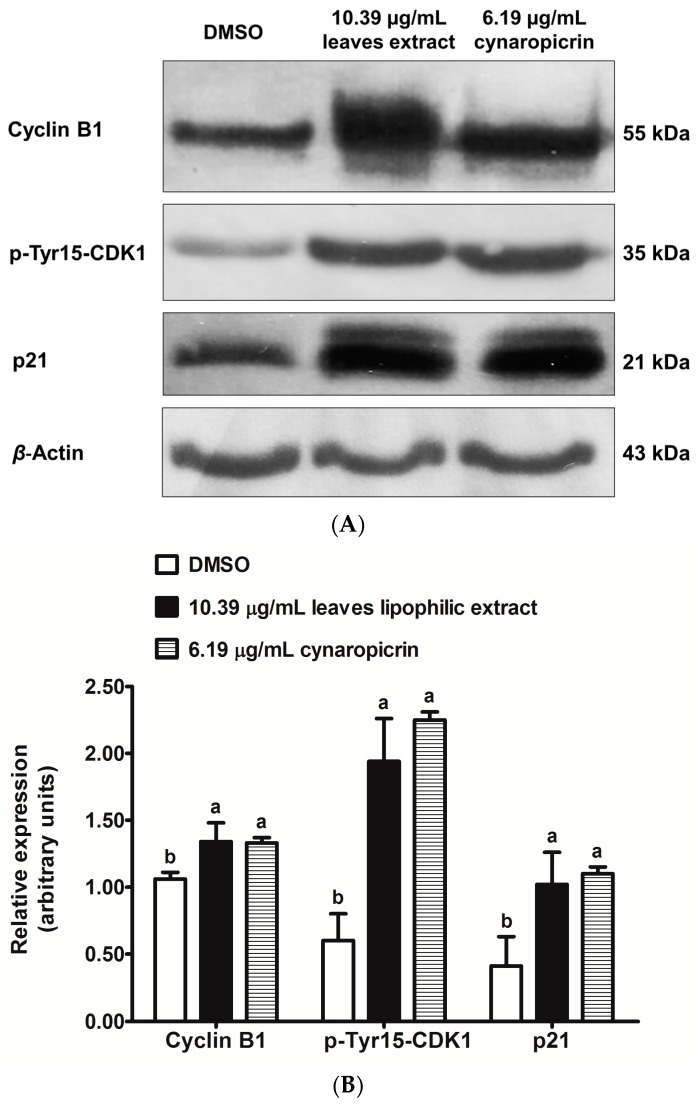
Western blot analysis of cyclin B1, p-Tyr15-CDK1 and p21 in MDA-MB-231 cells, after 48 h treatments with 10.39 μg/mL cultivated cardoon leaves lipophilic extract and 6.19 µg/mL cynaropicrin. DMSO (0.09% (*v*/*v*)) was used as solvent control. (**A**) Representative image of immunoblots; (**B**) Relative expression determined by analysis of band integrated density and normalization to β-actin. Each column and bar respectively represents the mean and the standard deviation of three independent experiments. Columns of each protein with different letters are statistically different (*p* < 0.05).

Protein expression levels of p21^Waf1/Cip1^ (hereafter referred as p21), phospho(Tyr15)-CDK1 (hereafter referred as p-Tyr15-CDK1) and cyclin B1 were analyzed in MDA-MB-231 cells, after 48 h treatments with cultivated cardoon leaves lipophilic extract (10.39 µg/mL) and cynaropicrin (6.19 µg/mL) (Figure 4A,B).

The relative expression levels of p21, p-Tyr15-CDK1 and cyclin B1 were significantly higher in MDA-MB-231 cells, after the treatment with cultivated cardoon leaves lipophilic extract (Figure 4B), in comparison with DMSO-treated control cells (*p* < 0.0071, *p* < 0.0003 and *p* < 0.0102 respectively). Furthermore, cynaropicrin also increased significantly the relative expression levels of these proteins in MDA-MB-231 cells, regarding to DMSO control cells (*p* < 0.0038, *p* < 0.0001 and *p* < 0.0119 respectively) (Figure 4B).

### 2.6. Effects of Cultivated Cardoon Leaves Lipophilic Extract and Cynaropicrin on Akt Signaling Pathway in MDA-MB-231 Cells

**Figure 5:**
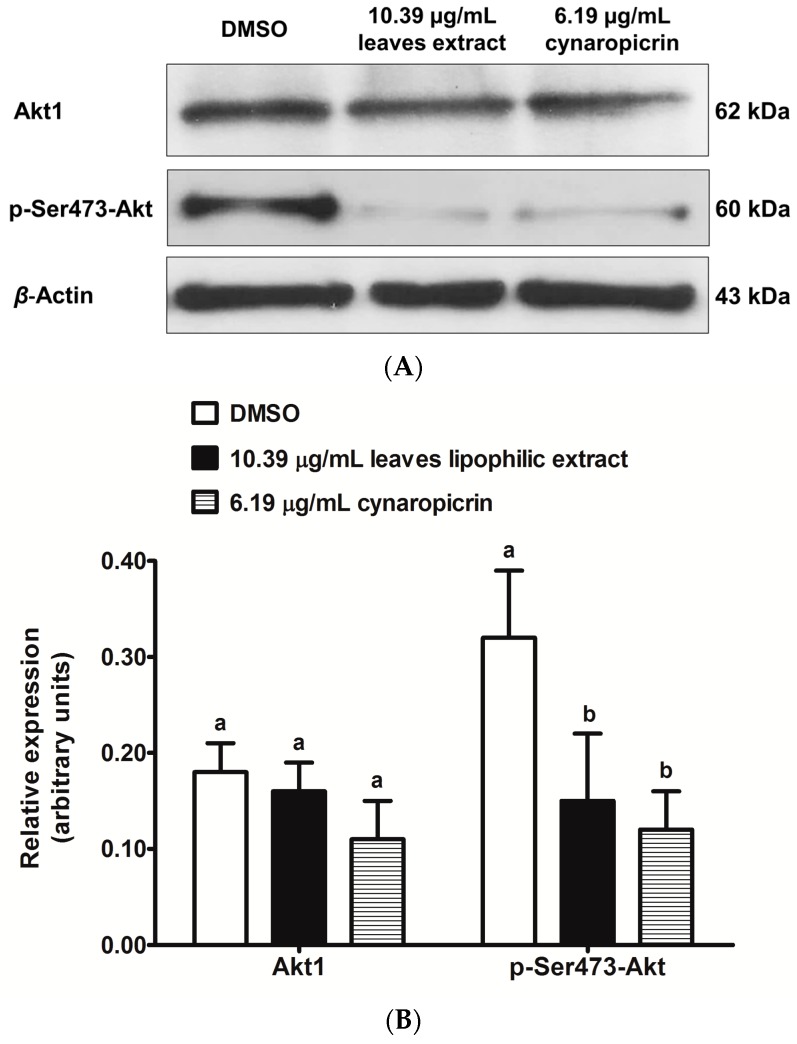
Western blot analysis of Akt1 and p-Ser473-Akt in MDA-MB-231 cells, after 48 h treatments with 10.39 μg/mL cultivated cardoon leaves lipophilic extract and 6.19 µg/mL cynaropicrin. DMSO (0.09% (*v*/*v*)) was used as solvent control. (**A**) Representative image of immunoblots; (**B**) Relative expression determined by analysis of band integrated density and normalization to β-actin. Each column and bar respectively represents the mean and the standard deviation of three independent experiments. Columns of each protein with different letters are statistically different (*p* < 0.05).

In order to better understand the regulating roles of cultivated cardoon leaves lipophilic extract and cynaropicrin in Akt signaling pathway [22], we evaluated the protein expression levels of phospho(Ser473)-Akt (hereafter referred as p-Ser473-Akt) and Akt1 in MDA-MB-231 cells, after 48 h (Figure 5A,B).

Cultivated cardoon leaves lipophilic extract and cynaropicrin treatments significantly decreased the relative expression of p-Ser473-Akt in MDA-MB-231 cells, compared to DMSO-treated control cells (*p* < 0.0164 and *p* < 0.0074 respectively) (Figure 5B). This finding corroborates with our previous data [22]. Cultivated cardoon leaves lipophilic extract and cynaropicrin did not significantly change the relative expression of Akt1 (*p* > 0.05) (Figure 5B). Further work must be done to gain more knowledge about the effects of cultivated cardoon leaves lipophilic extract and cynaropicrin in the PI3K/Akt/mTOR pathway.

## 3. Discussion

Currently, without targeted treatment, TNBC represents an extremely aggressive and highly refractory subtype of human breast cancer. As a diet rich in vegetables and fruits has been related to a reduced breast cancer incidence, due in part to the presence of bioactive secondary metabolites [4,5,6,7], our research group has sought the antiproliferative potential of natural sesquiterpene lactones [22] and pentacyclic triterpenes [24] as phytotherapeutics against TNBC. Given the high abundance of terpenes in *C. cardunculus* L. var. *altilis* (DC) [21], the present study highlights the in vitro antiproliferative effects of cultivated cardoon leaves and florets lipophilic extracts, as well as of the respective major extract component, i.e., cynaropicrin and taraxasteryl acetate, against TNBC MDA-MB-231 cells.

Our results denoted that leaves lipophilic extract was much more active than florets lipophilic extract in inhibiting the cellular viability of MDA-MB-231 cells for 48 h (see Table 1). Cynaropicrin also prevented the MDA-MB-231 cell growth for 48 h (IC_50_ 6.19 µg/mL), as earlier revealed [22]. Previously, this compound had also suppressed the in vitro cellular proliferation of another breast cancer subtype, i.e., MCF-7 cells (ER positive) (IC_50_ 1.10 µg/mL) [25]. The α,β-unsaturated ketone moiety probably plays an important role in the cytotoxicity of cynaropicrin, since it can react with biological nucleophiles, such as thiol groups in protein cysteine residues, via Michael addition [26,27]. Taking into account the considerably high content of cynaropicrin (455.2 mg/g extract), this compound may be the main one responsible for cultivated cardoon leaves lipophilic extract suppressive action, against MDA-MB-231 cell growth (IC_50_ 10.39 µg/mL). Actually, cultivated cardoon leaves lipophilic extract and cynaropicrin IC_50_ values (see Table 1), when expressed as µg/mL, were not statistically different (*p* > 0.05).

We then observed that cultivated cardoon leaves lipophilic extract also diminished the cellular viability of human breast epithelial MCF-10A cells for 48 h (IC_50_ 11.29 µg/mL). Despite IC_50_ values for MDA-MB-231 and MCF-10A cells were not significantly different (*p* > 0.05), the insights obtained in this work are sufficiently promising to continue the investigation on the pharmacological potential of cultivated cardoon leaves lipophilic extract and main extract components, in the scope of natural-based therapy against TNBC. Regarding to cynaropicrin, Cho, et al. [28] had reported that this compound reduced the 48 h-cell viability of human skin fibroblast Detroit 551 cells (IC_50_ 9.11 µg/mL).

With respect to taraxasteryl acetate, it was not possible to determine its IC_50_ value (>2.34 µg/mL), making difficult a comparison with cynaropicrin. Lhinhatrakool, et al. [29] had also noted that taraxasteryl acetate did not suppress the in vitro cellular viability of a breast cancer cell line, at 20.00 µg/mL.

We next assessed the capacity of cultivated cardoon leaves lipophilic extract (IC_50_ 10.39 µg/mL) and cynaropicrin (IC_50_ 6.19 µg/mL) to inhibit the formation of MDA-MB-231 cells colonies, through the anchorage-independent growth assay. Leaves lipophilic extract reduced the number and size of MDA-MB-231 cells colonies, over 14 days (see Figure 2), in a much more effective way than cynaropicrin. To the best of our knowledge, these effects of cultivated cardoon leaves lipophilic extract and cynaropicrin were proven here, for the first time. Rao, et al. [30] had similarly evidenced that MDA-MB-231 cells lost their ability to generate colonies under the treatment with the sesquiterpene lactone antrocin, isolated from the medicinal mushroom *Antrodia camphorata*.

Considering the suppressive action of cultivated cardoon leaves lipophilic extract on MDA-MB-231 cellular viability, we further addressed its biochemical mechanisms. Thus, we researched the hypothesis of apoptosis, by examining caspase-3 activity (see Figure 3). Caspase-3 is essential in the execution phase of apoptosis, as it specifically activates Caspase Activated DNAse, leading to chromosomal DNA degradation and chromatin condensation [31]. Caspase-3 activity was slightly higher in MDA-MB-231 cells, after 48 h treatment with leaves lipophilic extract (10.39 µg/mL). On the other hand, pure cynaropicrin (6.19 µg/mL) did not affect caspase-3 activity. Previous studies had reported that cynaropicrin induced apoptosis in other human tumor cell lines, namely gastric adenocarcinoma AGS [32] and leukocyte U937 cells [28]. Until now, there is no study that has reported the in vitro apoptotic effect of cynaropicrin in human breast cancer cells. Nonetheless, more details will be required to know whether cultivated cardoon leaves lipophilic extract and cynaropicrin exert apoptotic activity in MDA-MB-231 cells.

The hypothesis of cell death by cell cycle arrest was assessed by analyzing the MDA-MB-231 cells distribution through G0/G1, S and G2 phases, via flow cytometry, after 48 h-incubation (see Table 2). Cultivated cardoon leaves lipophilic extract (10.39 µg/mL) and cynaropicrin (6.19 µg/mL) arrested MDA-MB-231 cells at G2 phase, being in tune with our preliminary data [22]. Nonetheless, leaves lipophilic extract was more effective than cynaropicrin in increasing MDA-MB-231 cell percentage at G2 phase. In a previous work, Kang, et al. [32] had described that cynaropicrin arrested cell cycle of human gastric AGS cells at G2 phase.

Thereafter, we studied the relative expression levels of three known markers of G2/mitosis checkpoint, namely p21, p-Tyr15-CDK1, and cyclin B1 (see Figure 4). p21 suppresses the kinase activity of CDK2 bonded to cyclin E and cyclin A in G1/S, and of CDK1-2/cyclin A and CDK1/cyclin B complexes activities required for G2/mitosis [33,34]. p21 also mediates the nuclear retention of mitosis promoting factor (CDK1/cyclin B1), impeding its activation via CDK1 dephosphorylation at Thr14 and Tyr15 by Cdc25 fosfatase [35]. The activation of mitosis promoting factor is truly decisive for the transition of mammalian cells from G2 to mitosis. In what concerns to sesquiterpene lactones effects on G2/mitosis checkpoint markers, Rozenblat, et al. [36] had demonstrated that tomentosin and inuviscolide increased the protein levels of p21, p-Tyr15-Thr14-CDK1, and cyclin B1 in G2 arrested human melanoma SK-28 cells. We proved here for the first time that cultivated cardoon leaves lipophilic extract and cynaropicrin upregulated p21 protein level, and induced accumulation of p-Tyr15-CDK1 and cyclin B1 protein levels in MDA-MB-231 cells. These data may be accordingly associated with the G2 cell cycle arrest.

Akt has an important role in glucose metabolism, survival, cell proliferation, and programmed cell death [37,38]. Phosphorylated Akt (active form) frequently occurs in several types of cancer cells [37]. We had previously noticed that cultivated cardoon leaves dichloromethane extract and cynaropicrin regulated Akt phosphorylation in MDA-MB-231 cells [22]. In fact, both downregulated p-Ser473-Akt protein level, without changing total Akt1 protein level (see Figure 5). Thus, decreased p-Ser473-Akt protein level was not influenced by variations in Akt1 protein level. According to Rao, et al. [30], antrocin had also caused downregulation of p-Ser473-Akt protein level in MDA-MB-231 cells, without changing the total Akt protein level. Our data suggest that Akt signaling downregulation may be involved with antiproliferative effects of cultivated cardoon leaves lipophilic extract and cynaropicrin on MDA-MB-231 cells. However, future research will be needed to find out more information about their mechanism in the PI3K/Akt/mTOR pathway.

The inhibitory regulation of cultivated cardoon leaves lipophilic extract on MDA-MB-231 cell growth, shown in this work, may be related to the presence of cynaropicrin, but the possibility of other extract component(s) playing some relevant/synergic role should be further investigated. In the near future, the chemopreventive versus chemotherapeutic, or both, potential of cynaropicrin and cultivated cardoon leaves lipophilic extract should also be studied, using TNBC as model. According to the literature, other sesquiterpene lactones, such as parthenolide, have been described, so far with a chemopreventive role against human oral and renal cancer cell lines [39,40]. However, other studies had demonstrated a dual effect, as in the case of dehydrocostuslactone, a guaiane-type sesquiterpene lactone, which has been proven to be an inhibitor of human umbilical vein endothelial cells proliferation, and the in vivo data showed an anti-angiogenic potential, through Akt/glycogen synthase kinase-3β (GSK-3β) and mTOR pathways [41].

In summary, promising insights were provided in this work about the antiproliferative potential of cultivated cardoon leaves lipophilic extract and its major component cynaropicrin, against MDA-MB-231 cells, importantly contributing to the research of phytochemical-based therapy on TNBC.

## 4. Materials and Methods

### 4.1. Materials

Dichloromethane (p.a. ≥99% purity) was supplied by Fischer Scientific (Pittsburgh, PA, USA). Dulbecco’s modified Eagle’s medium (DMEM) 4.5 g/L glucose and l-glutamine, fetal bovine serum (FBS), and trypsin (5 g/L)-EDTA (2 g/L) were supplied by Lonza (Verviers, Belgium). Penicillin (10,000 units/mL)-streptomycin (10 mg/mL) mixture was provided by BioWest (Nuaillé, France). Cynaropicrin (≥97.5% purity) was purchased from Extrasynthese (Genay Cedex, France). Taraxasteryl acetate (≥99.2% purity) was provided by Avachem Scientific (San Antonio, TX, USA). DMSO cell culture grade was obtained from Applichem (Gatersleben, Germany). 3-(4,5-dimethylthiazol-2-yl)-2,5-diphenyltetrazolium bromide (MTT) and propidium iodide was purchased from Calbiochem (San Diego, CA, USA). DMEM F12, horse serum, epidermal growth factor (EGF) (≥90% purity), hydrocortisone (≥98% purity), cholera toxin (≈95% purity), insulin, agar, *p*-iodonitrotetrazolium violet, bovine serum albumin (BSA) (≥96% purity), and RNase were obtained from Sigma Chemicals Co. (Madrid, Spain). Caspase-3/CPP32 colorimetric protease assay was provided by Invitrogen (Camarillo, CA, USA). Anti-mouse horseradish peroxidase-conjugated secondary (No. 7076), p21 (No. 2946), cyclin B1 (No. 4135), and p-Ser473-Akt (No. 4051) antibodies were supplied by Cell Signaling Technology (Danvers, MA, USA). Donkey anti-goat horseradish peroxidase-conjugated secondary (sc-2020), Akt1 (sc-1618) and β-actin (sc-1616) antibodies were obtained from Santa Cruz Biotechnology (Dallas, TX, USA). p-Tyr15-CDK1 (no. 612306) antibody was purchased from BD Biosciences (San Jose, CA, USA). ECL reagents were provided by GE Healthcare Life Sciences (Buckinghamshire, UK).

### 4.2. Extracts Preparation and Chemical Characterization

*C. cardunculus* L. var. *altilis* (DC) (Asteraceae) leaves and florets were collected during the flowering stage, in June 2010 at the Experimental Center of the School of Agriculture (37°59′14.25″ N, 7°55′59.64″ W) from Instituto Politécnico de Beja, Beja, South Portugal. The plant materials were authenticated by the authors, and preserved at −20 °C until extraction. These materials were stored at Centro de Biotecnologia Agrícola e Agro-Alimentar do Alentejo, Beja, Portugal.

Before extraction, cultivated cardoon samples were freeze-dried and ground to a granulometry of 40–60 mesh. Leaves and florets were Soxhlet extracted with dichloromethane, as described elsewhere [21].

Lipophilic extracts were analyzed by gas chromatography-mass spectrometry, as previously reported [21,22]. Leaves and florets lipophilic extracts contained pentacyclic triterpenes (38.5 ± 0.3 and 320.6 ± 80.7 mg/g extract, respectively); sterols (3.4 ± 0.2 mg/g; 8.8 ± 4.4 mg/g) and fatty acids (2.5 ± 0.4 mg/g; 14.4 ± 0.4 mg/g). Sesquiterpene lactones were only detected in leaves lipophilic extract (484.9 ± 15.8 mg/g). The most abundant compounds of leaves and florets lipophilic extracts were cynaropicrin (455.2 ± 14.7 mg/g) and taraxasteryl acetate (93.4 ± 14.1 mg/g), respectively.

### 4.3. Cell Culture

MDA-MB-231 and MCF-10A cells were obtained from ATCC (Manassas, VA, USA). MDA-MB-231 cells were grown in DMEM supplemented with 10% (*v*/*v*) heat-inactivated FBS and 1% (*v*/*v*) penicillin-streptomycin mixture, while MCF-10A cells were cultured in DMEM F12 supplemented with 5% (*v*/*v*) horse serum, 20 ng/mL EGF, 0.5 mg/mL hydrocortisone, 100 ng/mL cholera toxin, 10 µg/mL insulin, and 1% (*v*/*v*) penicillin-streptomycin mixture. Cell cultures were maintained at 37 °C in a 5% CO_2_ humidified atmosphere (C150, Binder GmbH, Tuttlingen, Germany). Before confluence, cells were washed with phosphate buffered saline (PBS), collected following trypsinization with trypsin (0.5 g/L)-EDTA (0.2 g/L) solution and suspended in fresh growth medium before plating.

### 4.4. Cellular Viability by MTT Assay

Stock solutions of cultivated cardoon lipophilic extracts, cynaropicrin and taraxasteryl acetate, were prepared in DMSO. Cells were seeded in 96-well plates at 2 × 10^5^ cells/mL density, and incubated for 24 h at 37 °C. Then, cells were treated with leaves and florets lipophilic extracts (1–500 µg/mL), cynaropicrin (0.03–52.0 µg/mL or 0.1–150.0 µM) and taraxasteryl acetate (0.05–2.34 µg/mL or 0.1–5.0 µM) for 48 h. Solvent control cells received DMSO (<1% (*v*/*v*)). Cellular viability was determined using the MTT assay [42]. Therefore, cells were incubated with 20 μL, per well, of MTT stock solution (final concentration 0.5 mg/mL) in PBS, followed by incubation for 4 h at 37 °C. Medium was then discarded, and formazan crystals were solubilized in 100 µL of DMSO/ethanol (1:1) solution. The absorbance was read against a blank (DMSO/ethanol (1:1)) at 570 nm, using the Multiskan^TM^ FC microplate ultraviolet-visible spectrometer (Thermo Scientific, Waltham, MA, USA). The same experimental procedure was performed to test the effect of leaves lipophilic extract in MCF-10A cellular viability for 48 h. Cellular viability percentage was determined in relation to DMSO control. The IC_50_, defined as the sample concentration necessary to cause 50% inhibition of cellular viability, was calculated by plotting the percentage of cellular viability against the sample concentration logarithm. Three independent experiments were performed in triplicate.

### 4.5. Soft Agar Colony Formation Assay

MDA-MB-231 cells (9,200 cells per well) were seeded in 0.3% agar on top of 0.5% pre-solidified agar, in a 12-well plate. Each treatment was performed in triplicate. After 24 h of incubation at 37 °C, cells were treated with cultivated cardoon leaves lipophilic extract (IC_50_ 10.39 µg/mL) and cynaropicrin (IC_50_ 6.19 µg/mL). Solvent control cells received DMSO (0.09% (*v*/*v*)). Every other day, agar layers were supplemented with the above-mentioned samples. After 14 consecutive days, cells were incubated overnight with 0.1% *p*-iodonitrotetrazolium violet at 37 °C. Colonies were thereafter observed using an inverted microscope (Motic, Xiamen, China) at the 40× magnification, and four fields of each well were photographed using Moticam 2500 camera (Motic, Xiamen, China). Images were processed using the Motic Images Plus 2.0 software (Motic, Xiamen, China).

### 4.6. Caspase-3 Activity Analysis

Caspase-3 activity was assessed following the manufacturer’s protocol of caspase-3/CPP32 colorimetric protease assay. DEVD-*p*NA is composed by the synthetic tetrapeptide substrate Asp-Glue-Val-Asp (the upstream amino acid sequence of caspase-3 cleavage site in poly(adenosine diphosphate-ribose) polymerase), and tailed with *p*-nitroanilide (*p*-NA) chromophore. MDA-MB-231 cells were seeded at 5 × 10^5^ cells/mL, and after a 24 h-incubation at 37 °C, cells were exposed to cultivated cardoon leaves lipophilic extract (10.39 µg/mL) and cynaropicrin (6.19 µg/mL). Solvent control cells were incubated with 0.09% (*v*/*v*) DMSO. After 48 h, cells were collected and centrifuged at 769× *g* for 5 min. The cell pellet (5 × 10^6^ cells) was resuspended in 50 µL of chilled cell lysis buffer and kept on ice for 10 min. After centrifugation at 10,000× *g* for 1 min at 4 °C, supernatant (cytosol) was collected for caspase-3 activity analysis. Total protein concentration was assayed according to Lowry method [43], and BSA was used as protein standard. Cytosol samples (150 µg protein) were mixed with 50 µL of 2× reaction buffer (10 mM dithiothreitol), and 5 µL of 4 mM DEVD-*p*NA. After 2 h of incubation at 37 °C in the dark, the absorbance of *p*-NA was read at 405 nm against a blank (without sample), utilizing the Multiskan™ FC microplate ultraviolet-visible spectrometer (Thermo Scientific, Waltham, MA, USA). Three independent experiments were carried out.

### 4.7. Cell Distribution at G1, S, and G2 Cell Cycle Phases by Flow Cytometry

MDA-MB-231 cells were cultured in 6-well plates at a density of 4 × 10^5^ cells/mL, for 24 h at 37 °C. Then, cells were treated with cultivated cardoon leaves lipophilic extract (10.39 µg/mL) and cynaropicrin (6.19 µg/mL). Solvent control cells received 0.09% (*v*/*v*) DMSO. After the 48 h of incubation at 37 °C, cells were collected, PBS washed, and fixed with 85% cold ethanol. Cell pellets were collected after centrifugation at 300× *g* for 5 min at 4 °C, and resuspended in PBS. Cells were then incubated with 50 µg/mL RNase and 50 µg/mL propidium iodide staining solution, for 20 min at room temperature in the dark. Propidium iodide-stained cells were analyzed in the Beckman-Coulter^®^ EPICS-XL flow cytometer (Beckman-Coulter^®^, Brea, CA, USA) equipped with an air-cooled argon-ion laser (15 mW, 488 nm). Data were obtained using the SYSTEM II software (version 3.0 Beckman-Coulter^®^, Brea, CA, USA), in which at least 5000 nuclei per sample were acquired. Analysis of cell cycle distribution was carried out with the FlowJo software (Tree Star, Ashland, OR, USA). Four replicates were performed for each treatment.

### 4.8. Western Blotting

MDA-MB-231 cells were plated at 5 × 10^5^ cells/mL, and incubated for 24 h at 37 °C. The medium was thereafter changed to medium containing cultivated cardoon leaves lipophilic extract (10.39 µg/mL) and cynaropicrin (6.19 µg/mL). Solvent control cells were incubated with 0.09% (*v*/*v*) DMSO. After 48 h of incubation at 37 °C, cells were scraped, washed in cold PBS, and centrifuged at 492× *g* for 3 min at 4 °C. This procedure was repeated two more times. Cells were then lysed with RIPA buffer (1% NP-40 in 150 mM NaCl, 50 mM Tris-HCl (pH 8), 2 mM EDTA), containing 1 mM phenylmethylsulfonylfluoride, phosphatase inhibitors (20 mM NaF, 20 mM Na_2_V_3_O_4_), and protease inhibitor cocktail (Roche, Mannheim, Germany), for 10 min at 4 °C. Cell lysates were centrifuged at 24,104× *g* for 10 min at 4 °C. Supernatants were collected and total protein concentrations were quantified based on the Lowry method [43], using BSA as the protein standard. Cell lysates (25–40 µg protein) were electrophoresed on sodium dodecyl sulfate 10% polyacrylamide gel, and then transferred onto poly(vinylidene difluoride) (PVDF) membranes (Amersham Biosciences, Buckinghamshire, UK). PVDF membranes were blocked with 5% (*w*/*v*) of nonfat dry milk at room temperature for 1 h, and incubated overnight at 4 °C with a primary antibody against p21 (1:2000); p-Tyr15-CDK1 (1:250); cyclin B1 (1:1000); p-Ser473-Akt (1:1000); Akt1 (1:200); and β-actin (1:300). Bands were visualized by chemiluminescence using appropriate horseradish peroxidase-conjugated secondary antibodies, and developed with ECL reagents (GE Healthcare Life Sciences, Amersham Biosciences, Buckinghamshire, UK) according to the manufacturer’s instructions. Three independent experiments were performed for each treatment. The relative expression was determined by densitometry, with normalization to β-actin.

### 4.9. Statistical Analysis

All parameters measured were analyzed using the PROC GLM option of SAS (SAS Institute Inc., Cary, NC, USA). Where differences existed, the source of the differences at *p* < 0.05 of significance level was identified by all pairwise multiple comparison procedures via the Tukey’s test.

## 5. Conclusions

The present study demonstrates important data about the in vitro antiproliferative potential of *C. cardunculus* L. var. *altilis* (DC) leaves and florets lipophilic extracts, and their major compounds, i.e., cynaropicrin and taraxasteryl acetate, against TNBC MDA-MB-231 cells, a highly refractory human cancer. Leaves lipophilic extract was more active than florets lipophilic extract in inhibiting MDA-MB-231 cell growth for 48 h. Moreover, cultivated cardoon leaves lipophilic extract and cynaropicrin increased the relative expression levels of important G2/mitosis checkpoint proteins, namely p21^Waf1/Cip1^, p-Tyr15-CDK1, and cyclin B1, which may be related to the G2 cell cycle arrest. Both also downregulated the p-Ser473-Akt protein level, without affecting the total Akt1 protein level. Additionally, cultivated cardoon leaves lipophilic extract decreased caspase-3 activity, but the apoptosis hypothesis needs to be further studied with more detail. Cultivated cardoon leaves lipophilic extract and cynaropicrin also suppressed the MDA-MB-231 cell capacity to generate colonies in a three-dimensional environment. Considering its high content (455.2 mg/g extract), cynaropicrin was, most probably, the major component that contributed in the antiproliferative action of cultivated cardoon leaves lipophilic extract. Nonetheless, other extract component(s) may have also been involved.

Based on the overall results of this work, it is important to proceed with the research about the anticancer potential of cultivated cardoon leaves lipophilic fraction and cynaropicrin toward novel or complementary natural-based therapeutic approaches against human TNBC.

## Figures and Tables

**Table 1:** IC_50_ values of cultivated cardoon lipophilic extracts and cynaropicrin on the 48 h cellular viability of MDA-MB-231 cells, by using the MTT assay.

Cultivated Cardoon Extract/Pure Compound	IC_50_ for Antiproliferative Activity (µg/mL) ^1^
Leaves lipophilic extract	10.39 ± 0.41 ^b^
Florets lipophilic extract	315.22 ± 67.88 ^a^
Cynaropicrin	6.19 ± 0.57 ^b^

^1^ Each value is expressed as mean ± standard deviation. Three independent experiments were carried out. Means marked with different letters are statistically different (*p* < 0.05).

**Table 2:** Cell cycle phases distribution of MDA-MB-231 cells, after 48 h treatments with cultivated cardoon leaves lipophilic extract (10.39 µg/mL) and cynaropicrin (6.19 µg/mL). Solvent control cells received DMSO (0.09% (*v*/*v*)).

Group	MDA-MB-231 Cells (%) ^1^		
	G0/G1	S	G2
DMSO	42.5 ± 2.8 ^a^	45.0 ± 2.2 ^a^	12.6 ± 3.0 ^c^
10.39 µg/mL leaves lipophilic extract	15.8 ± 3.1 ^b^	24.8 ± 2.1 ^b^	59.5 ± 1.1 ^a^
6.19 µg/mL cynaropicrin	41.2 ± 3.8 ^a^	21.0 ± 3.7 ^b^	37.8 ± 3.1 ^b^

^1^ Each value is expressed as mean ± standard deviation. Four replicates were carried out. Means marked with different letters within the same column are statistically different (*p* < 0.05).
